# Reliability of Rodent and Rabbit Models in Preeclampsia Research

**DOI:** 10.3390/ijms232214344

**Published:** 2022-11-18

**Authors:** Agata Sakowicz, Michalina Bralewska, Piotr Kamola, Tadeusz Pietrucha

**Affiliations:** 1 Department of Medical Biotechnology, Medical University of Lodz, 90-752 Lodz, Poland; 2 Department of Haemostasis and Haemostatic Disorders, Biomedical Sciences, Medical University of Lodz, Mazowiecka 6/8, 92-215 Lodz, Poland

**Keywords:** mouse, placenta, preeclampsia, rabbit, rat, rodents, trophoblast

## Abstract

In vivo studies on the pathology of gestation, including preeclampsia, often use small mammals such as rabbits or rodents, i.e., mice, rats, hamsters, and guinea pigs. The key advantage of these animals is their short reproductive cycle; in addition, similar to humans, they also develop a haemochorial placenta and present a similar transformation of maternal spiral arteries. Interestingly, pregnant dams also demonstrate a similar reaction to inflammatory factors and placentally derived antiangiogenic factors, i.e., soluble fms-like tyrosine kinase 1 (sFlt-1) or soluble endoglin-1 (sEng), as preeclamptic women: all animals present an increase in blood pressure and usually proteinuria. These constitute the classical duet that allows for the recognition of preeclampsia. However, the time of initiation of maternal vessel remodelling and the depth of trophoblast invasion differs between rabbits, rodents, and humans. Unfortunately, at present, no known animal replicates a human pregnancy exactly, and hence, the use of rabbit and rodent models is restricted to the investigation of individual aspects of human gestation only. This article compares the process of placentation in rodents, rabbits, and humans, which should be considered when planning experiments on preeclampsia; these aspects might determine the success, or failure, of the study. The report also reviews the rodent and rabbit models used to investigate certain aspects of the pathomechanism of human preeclampsia, especially those related to incorrect trophoblast invasion, placental hypoxia, inflammation, or maternal endothelial dysfunction.

## 1. Introduction

Preeclampsia is a disorder associated with pregnancy and entails a high risk of maternal and foetal mortality and morbidity. Its prevalence is estimated to be 5–8% of all expectant mothers and typically manifests after week 20 of gestation. The sudden appearance of hypertension (i.e., >140 mmHg systolic or >90 mmHg diastolic) in previously normotensive women accompanied by proteinuria (i.e., >300 mg for 24 h or at least 2+ on a dipstick) typically indicates the onset of preeclampsia. However, the presence of hypertension complicated by at least one of the following symptoms, viz. serum creatinine level > 1 mg/dL, elevated transaminase levels, thrombocytopenia, haemolysis, neurological disorders, or uteroplacental dysfunction (i.e., foetal growth restriction), is also sufficient to identify preeclampsia [1,2].

Although the clinical symptoms of preeclampsia arise in the latter half of pregnancy, it is believed that the cause of the disease should be sought at the beginning of gestation. Shallow trophoblast invasion into the maternal spiral arteries disturbs their transformation into wider vessels [3], thus preventing access of the placental bed to oxygen and nutrients. This results in placental apoptosis and the appearance of various placental factors such as sFlt-1, arginase, or sEng in maternal circulation; these stimulate the maternal immunological response, as recognised by the presence of elevated levels of inflammatory factors, e.g., TNFα, IL-1, or IL-6 in the maternal blood [4]. As a consequence, oxidative stress and maternal endothelium dysfunction develop [5,6,7]. The endothelial cells downregulate the secretion of vessel relaxation substances, thus disturbing the regulation of vascular tone and increasing hypertension.

Although the factors appearing in maternal blood at various stages of preeclamptic pregnancy are understood, little is known about the mechanisms influencing improper trophoblast invasion. A more complete understanding of the pathomechanism of preeclampsia will undoubtedly support the development of new strategies for the prevention and treatment of preeclampsia; currently, the only way to help mother and child is caesarean section. To better understand the molecular mechanisms involved in the condition, studies should move beyond the factors appearing in maternal blood to include those present in placental tissue, especially in the tissue developing in the first trimester of pregnancy, i.e., when the source of the later pathological processes starts to appear.

However, due to the legal and ethical matters preventing the use of human placentas obtained at various stages of pregnancy, animal models are regarded as the best chance of solving the mystery of preeclampsia. When choosing a suitable animal model for this condition, the researcher should consider the potential etiological factors active in the analysed stage of human gestation; indeed, a range of genetic, morphological, hormonal, or immunological influences might be engaged at the same time in the pathomechanism of preeclampsia. Therefore, it is very important to employ an animal model that corresponds as closely to the analysed period of human gestation as possible.

This paper reviews the current state of knowledge regarding the physiology of rodent and rabbit pregnancy, as these are the most common animal models used for preeclampsia. Their properties will be compared with those of similar stages in human gestation. Particular attention will be also be paid to studies using animals to investigate incorrect trophoblast invasion and placenta formation, as these processes are linked with the first trimester of gestation, which is when preeclampsia is believed to be initiated. Additionally, the review will examine animal models used to examine the factors that can induce maternal inflammation, endothelium dysfunction, and hypertension when secreted from the ischaemic placenta into maternal circulation.

## 2. A Comparison of the Physiology of Pregnancy in Humans and Small Mammals

### 2.1. Implantation

The process of implantation leading to the infiltration of trophoectoderm into the maternal decidua consists of apposition, attachment, and the adhesion of the blastocyst to the luminal epithelium of the uterus. All of these stages are typical for humans and small mammals, including rabbits and rodents. The success of this process depends on the correct preparation of the endometrium to interface with the blastocyst. This receptivity of maternal tissue for implantation only lasts for a few days or hours, depending on the species. In humans, the uterus is prepared for contact with the blastocyst before ovulation, thus allowing it to become completely receptive a few days after ovulation. The uterus is receptive during each menstrual cycle, occurring five to nine days post ovulation, regardless of the presence or absence of an implanting blastocyst [8]. In rodents, the uterus only demonstrates changes in lining after effective fertilization, i.e., while awaiting the blastocysts. Implantation chambers, known as mucosal crypts, appear inside the mucosa along the uterine horn, and each one permits only a single blastocyst [9].

**Figure 1:**
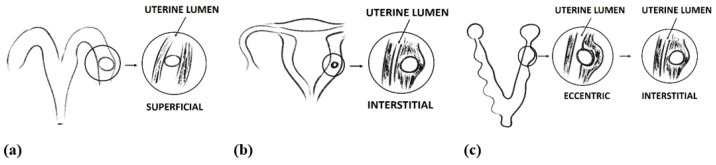
Models of implantation: (**a**) superficial in rabbit, (**b**) interstitial in human, and (**c**) eccentric and interstitial in rodents.

Generally, in rodents, the inner cell mass of the blastocyst is orientated towards the mesometrium; however, in humans, the mass can migrate along the inside of the blastocyst to orient itself towards the endometrium [10,11]. The receptivity of the endometrium to the blastocyst varies slightly between rodents; it is initiated between days 4 and 5 in golden hamsters and mice, between days 5 and 6 in laboratory rats, and on days 7–8 in guinea pigs [11,12,13]. For rabbits, this period varies according to the region of the uterus, i.e., on days 7–8 for the antimesometrial region and on days 8–9 for the mesometrial region of the uterus. Indeed, similar to sheep, pigs, and monkeys, rabbits demonstrate a central (superficial) type of implantation, where obplacental implantation occurs in the antimesometrial region of the uterus, and placental implantation occurs in the mesometrial region [14,15]; thus, the chorionic sac remains in contact with the uterine lumen (Figure 1a) [9,15]. Humans and rodents present an interstitial model of implantation, in which the whole blastocyst implants deep inside the uterine wall, and the chorionic sac loses contact with the uterine lumen (Figure 1b); however, in rodents, the chorionic sac resides inside the pocket of uterine tissue during early gestation and remains in slight contact with the uterine lumen. Therefore, in rodents, early pregnancy is characterised by the eccentric model (Figure 1c) of implantation [9].

### 2.2. Decidualization

The process of implantation is strictly related to decidualization, i.e., the modification of certain features of the stromal cells resident in the maternal endometrium. These changes include modifications in the metabolism as well as the organisation and shape of the cells. In humans, this process occurs independently of the implanted blastocyst and begins spontaneously in the mild secretory phase of each menstrual cycle [16]; as such, the first transformed cells appear before pregnancy. In rodents, both hormonal and implantation signals are required to prepare the maternal tissue for further pregnancy [17]; thus, the first decidual cells, which are derived from transformed fibroblasts of the stroma, appear when the blastocysts reach the basal lamina layer of the uterus [11]. In humans, decidualization leads to the formation of three types of deciduae: decidua basalis, which is localised under the site of implantation; decidua capsularis, which overlies the developing foetus; and decidua vera, which pads the remaining part of the uterine cavity [10]. In rodents, decidualization is restricted to only the region surrounding the blastocyst, and the spaces between the implanted regions are not influenced by this process [11].

### 2.3. Trophoblast Invasion

**Figure 2:**
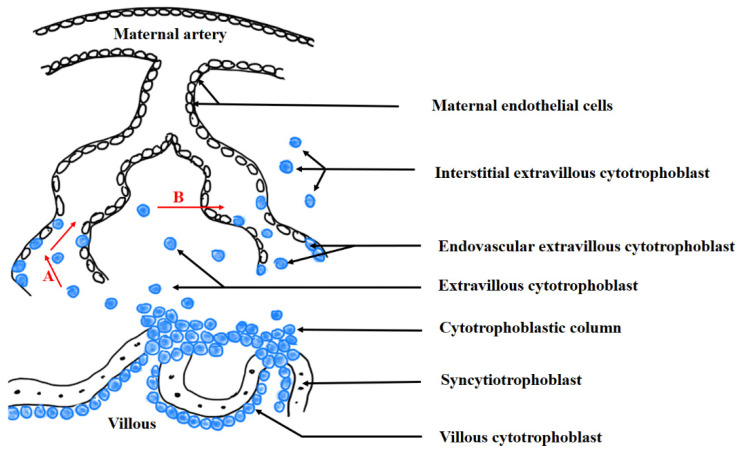
The process of trophoblast invasion into maternal uterine arteries. Red arrows indicate the direction of trophoblast invasion. A—extravasation; B—intravasation. Black arrows indicate the cells participating in the described process. The name of the cells is given on the right side of the black arrow. The blue circles present the cytotrophoblastic cells. The black circles present the maternal endothelial cells.

In humans, immediately after implantation, the endometrial stroma surrounds the implantation site, and the trophectodermal layer of the blastocyst generates the first miscellaneous trophoblast cells [18,19]. Following implantation, the cytotrophoblast cells developed from the trophoectodermal layer of the blastocysts start to transform into syncytiotrophoblasts (SYNs) and into extravillous trophoblasts (EVTs). The former are connected with the placental villi and typically express epithelial components such as E-cadherin, which is responsible for cell–cell adhesion, and α_6_ and β_4_ integrins. The creation of the extravillous trophoblast is related to the epithelial–mesenchymal transition (EMT). The cells start to reduce their production of E-cadherin and α_6_β_4_ and increase their production of α_5_β_1_ integrin; this is a classic marker of the mesenchymal cells localised at the end of the placental villi. In this location, the syncytiotrophoblast layer is absent, and cytotrophoblastic cell columns are formed. The cells building neighbouring columns become attached, creating the cytotrophoblast shell [20]. During gestation, the cells become detached from the column and migrate inside the uterine decidua, where they form the interstitial cytotrophoblasts. They demonstrate a mesenchymal phenotype with the expression of the specific heterodimer, α_1_β_1_, a receptor for the laminin and collagen present in the extracellular matrix of the uterus [21,22] and that is characteristic of extravillous cytotrophoblasts. These cytotrophoblasts are also characterised by the high expression of the metalloproteinases implicated in uterine invasion and with the unusual class 1b major histocompatibility antigen (HLA-G), which plays a role in evading the maternal immune reaction, thus protecting invading cells against the natural killer (NK) cells stationed in the uterine wall [23]. These extravillous cytotrophoblastic cells have high invasive potential and enable the transformation of maternal spiral arteries; they also form a cytotrophoblast plug that occludes maternal blood vessels. Interestingly, the maternal plasma can seep into the intervillous spaces through clefts created by the plug; however, maternal blood cells are prevented from entry when the plug is present [24,25]. This seal allows the maintenance of hypoxia, which supports the proliferative potential of extravillous trophoblastic cells and their invasion into the uterine spiral arteries. Once disconnected from the cytotrophoblast, these highly invasive cells, when disconnected from the cytotrophoblast shell, reach the maternal vessels by one of the following processes: (1) intravasation, in which extravillous cells known as interstitial cytotrophoblasts, which are localised inside the decidua, migrate through the arterial wall into the arterial lumen; in humans, this process starts before week 6 of gestation; (2) extravasation, in which the endovascular cytotrophoblast cells detach from the cytotrophoblast column and invade the distal part of the vessels directly via their lumen (Figure 2) [20,25,26,27].

Depending the type of the vessels undergoing trophoblast invasion, the endovascular trophoblast might be regarded as (1) an endovenous trophoblast, influencing the opening of maternal veins to allow the backflow of maternal blood from intervillous spaces of the placenta into maternal circulation, starting at approximately week 5 of gestation, or (2) an endoarterial trophoblast, migrating deep into maternal arteries and supporting their transformation beginning at about gestational week 7 or 8 [25,26]. The infiltration of maternal vessels by extravillous trophoblasts leads to the erosion of their smooth muscle and basement membrane as well as to the replacement of the maternal endothelial cells by the invading trophoblast. This process continues until the trophoblast reaches the distal fragments of the arteries located within the inner third of the myometrium [28,29].

In rodents, i.e., mice and rats, the cells of the extraembryonic ectoderm, a derivative of the trophectoderm layer of the blastocyst, differentiate into the chorionic ectoderm and ectoplacental cone [17,30]. The extraembryonic ectoderm contributes to the formation of the placental labyrinth, corresponding to the human villous trophoblast and including the lacunas where foetal–maternal nutrient and gas exchange takes place [17,30]. The latter layer forms the junctional zone, known as the basal zone or trophospondium [31] of the placenta, and it corresponds to the extravillous trophoblast in humans [17]. This zone consists of spongiotrophoblasts, glycogen, and trophoblastic giant cells [17,32,33]. Both the labyrinth and junctional zone exist on the foetal side and are separated from the maternal side, i.e., the decidua and mesomertial layer, by trophoblastic giant cells [30,34]. These cells are the main source of placental hormones and cytokines, and they might invade the maternal decidua, thus participating in the transformation of maternal spiral arteries [20,35]. Similar to humans, rats, hamsters, and guinea pigs use interstitial and endovascular trophoblasts for remodelling spiral arteries [31,36]. Similar to humans, in rodents, the epithelial–mesenchymal transition is followed by trophoblast invasion into the maternal uterine wall. This is related to the downregulation of E-cadherin, the upregulation of metalloproteinases such as MMP9 [20], and the elevated expression of heterodimer α1β1 [37]. In mice, the interstitial trophoblast is mainly responsible for the transformation of the maternal arteries, especially at the beginning of pregnancy, i.e., at about 8–12 gestational days (GD) [36,38,39]. Endovascular trophoblast invasion is observed following the establishment of the vascular maternal–foetal connection [40]. In rabbits, the replacement of maternal endothelial cells and thus the remodelling of spiral arteries is carried out by placental multinucleated giant cells [41].

The process of maternal artery transformation depends on the depth of trophoblast invasion and varies between species. The shallowest trophoblast invasion is observed in mice, in which the trophoblastic cells reach the lumen of the spiral arteries of the proximal part of the decidua [39,40]. Deep trophoblast invasion has been observed in rats and guinea pigs as well as in humans; in rats, the trophoblastic cells migrate into an artery localised in the distal part of the placenta, e.g., in the mesometrial triangle [20,39,42,43,44,45,46]. Little is known about the migration of giant cells in rabbits; however, they have been found to be present in the vessel wall, far from the deepest limit of the placenta labyrinth, i.e., in the mesometrial layer [47,48]. Hamsters demonstrate much deeper trophoblast invasion than humans, and the cells migrate far beyond the uterus [49].

Similar to humans, hamsters develop a plug inhibiting maternal blood inflow into the maternal sinusoid at the beginning of gestation [31,36,50]. This plug is created between days 8 and 12 of gestation by giant cells at the end of maternal spiral arteries localised in the basal plate layer [31,50]. Rats and rabbits do not form trophoblastic plugs; however, the maternal arterioles in the myometrium are constricted sufficiently to prevent the passage of blood cells but to allow maternal plasma to approach the site of constriction [51,52]. Utero-placental circulation is recognised as significant in rabbits at around gestational day 10 and in rats between days 12 and 15 [51,53]. Although mice and guinea pigs present similar times for maternal blood to pass into the placental sinuses as rats and rabbits, previous studies do not confirm the formation of a trophoblastic plug in the placentas of these animals [36,54,55,56]. The formation of a trophoblastic plug could be related to the initiation of maternal spiral artery transformation. While the initial changes occurring in the uterine arteries begin before pregnancy in humans [3], spiral artery formation begins at day 5 of gestation, i.e., on the day of implantation, in hamsters [57]. Mice, rats, guinea pigs, and rabbits begin to undergo maternal spiral artery transformation after implantation; however, this process appears a little earlier in rats and rabbits than in mice and guinea pigs [17,58,59,60].

It is possible that an earlier onset of spiral artery transformation might result in earlier contact between maternal blood and the trophoblasts. In such cases, the formation of a trophoblastic plug or the constriction of the maternal vessels might act as a protective mechanism against placental hyperoxia. It is obvious that placental hypoxia, which is observed at the beginning of each pregnancy, is desirable because it supports the process of angiogenesis and modulates the invasive and proliferative capacity of the trophoblast cells [44,61,62]. Moreover, low oxygen tension appears to modulate the differentiation of cytrotrophoblastic cells into the EVT lineage. Interestingly, the cells living in the cytotrophoblastic cell column present a high proliferative potential and can undergo the epithelial–mesenchymal transition; however, their invasiveness potential is low. When the EVT first makes contact with the maternal decidua, and thus with an environment that is a little more normoxic, the behaviour of the trophoblastic cells changes. They still present the potential for differentiation but also acquire an invasive phenotype. As a result, they move through the maternal tissues; all of this occurs along a steep oxygen gradient [28,63,64]. The process of invasion is supported by inflammation; the uterus is infiltrated by immunological cells such as natural killer cells (NK) or macrophages that secrete pro-inflammatory factors (e.g., TNFα, IL1, IL6, and IL8) and metalloproteinases. These degrade the extracellular matrix (e.g., MMP2 and MMP9), thus supporting the process of implantation, trophoblast invasion, and placentation [3].

Interestingly, the secretion of inflammatory factors by the maternal immunological cells situated in the decidua may be regulated by extravillous cytotrophoblastic cells. These cells present an unusual class 1b major histocompatibility antigen (HLA-G) on their surface; this is a ligand for the receptors KIR2DL4, known as CD158d, and ILT2, which are presented by uterine natural killer cells (uNK), macrophages, and decidual T cells [65,66]. The HLA-G-KIR2DL4 or HLA-G–ILT2 interactions rescue the cytotrophoblastic cells from cytolysis by uNK, constituting 70% of all leucocytes in the decidua during the first trimester, as well as T lymphocytes, constituting 5–15% of uterine white blood cells. In addition, it also promotes the survival of EVT cells and influences the formation of foetal–maternal immune tolerance, which is important during pregnancy [65,66]. The interaction between HLA-G and its receptors also facilitates foetal growth by stimulating uNK to secrete growth-promoting factors such as pleiotrophin, osteoglycin, and osteoponin [67]. Similar foetal growth-promoting factors are secreted by the CD49a^+^ Eomes^+^ subset of NK cells in rodents [67]. Moreover, HLA-G is implicated in uterine artery spiral remodelling. This process is essential for proper placentation and is initiated by uNK in both humans and rodents [17,20]. The HLA-G-KIR2DL4 interaction results in the senescence and cell cycle arrest of uNKs and eventually leads to their apoptosis. However, before programmed death, uNKs are metabolically very active and secrete a set of mediators termed the senescent-associated secretory phenotype (SASP), which include the pro-inflammatory cytokines, growth factors, and proteases that are implicated in both trophoblast invasion and maternal spiral remodelling [68]. Interestingly, although neither HLA-G nor KIR2DL4 is present in rodents, their uNKs and T lymphocytes present the KLRG1 receptor on their surface as a marker of their senescence; this indicates that both humans and small mammals use SASP mediators for ensuring a correct pregnancy outcome [68].

### 2.4. Maternal–Foetal Blood Barrier

Pregnancies based on haemochorial-type placentas, including those of humans, rodents, and rabbits, are characterised by trophoblast invasion into uterine tissue. This allows the remodelling of maternal spiral vessels and their transformation into wider ones with lower resistance. While maternal vessel transformation begins before pregnancy in humans, it is initiated at the beginning of gestation in rodents, e.g., at about day 5 in hamsters, 6.5 in rats, or 8–12 in mice [17,38,57]. It is believed that both maternal natural killer cells (NKs) and invasive trophoblastic cells play a role in the maternal transformation of maternal spiral arteries in human and rodents. The NK cells dominate in the first stage of the transformation, whereas the endovascular invasive trophoblastic cells play the key role in the second stage, in which they remodel all of the components of the maternal arteries, i.e., smooth muscle cells as well as basement membrane and maternal endothelial cells [17].

Following the transformation of the maternal vessels, the walls of the arteries become thin and distensible, and the arterial lumen widens to effectively supply the maternal blood to the spaces between the placental villi in humans or into trophoblast-lined lacunae (known also as canals or sinus) in rodents and rabbits [40,69,70]. In humans, together with endovascular trophoblast invasion and the transformation of maternal arteries, the trophoblastic plug is gradually degraded and completely removed from the maternal vessels at the end of the first trimester of gestation, i.e., about week twelve. This allows for the free inflow of maternal blood into the intervillous spaces.

In humans and guinea pigs, the maternal blood is separated from the foetal blood by only one layer, the syncytiotrophoblast; in some cases, this is accompanied by a slightly reduced cytotrophoblast; this type of placenta is known as hemimonochorial. In contrast, rabbits present a hemidichorial placenta, in which the trophoblast forms a double layer separating the foetal and maternal blood and comprising the cytotrophoblast and syncytiotrophoblast. The most complex structure separating maternal and foetal blood is found in the hemitrichorial placentas of mice and rats. In this type, the barrier is formed from three layers: syncytiotrophoblast I, syncytiotrophoblast II, and cytotrophoblasts [71].

In both humans and small rodents, the influx of maternal blood into the intervillous spaces increases the supply of oxygen and nutrients to the foetus and placenta [24,72]. As a result, the local oxygen tension increases to 40–60 mmHg, i.e., 5–8% O_2_, and this normoxic state for placental cells is maintained until the end of gestation [24,62].

## 3. Pathomechanism of Preeclampsia

Spontaneous preeclampsia appears in humans and in some higher apes [73]. Although it has been known about since ancient times, and even though knowledge about the pathomechanism of preeclampsia is constantly increasing, its precise nature remains unclear. A significant body of evidence indicates that preeclampsia is closely related to incorrect trophoblast invasion into the maternal decidua. This results in the incomplete transformation of uterine spiral arteries; the vessels lose their elasticity and dilatation capabilities, reducing the efficiency of maternal blood inflow into the placental sinuses. Interestingly, although gynecological societies recognise that early-onset and late-onset preeclampsia (before or after week 34 of gestation, respectively, and with or without foetal growth restrictions) have different etiologies, placentas obtained from early and late preeclamptic mothers present defective remodelling of the decidual and myometrial spiral arteries [74,75].

It remains unclear which factors inhibit trophoblast invasion. It has been proposed that an excessively strong inflammatory reaction may inhibit invasion by damaging the foetal–maternal interface [76,77]. As inflammation is often accompanied by hypoxia, the cells living in the cytotrophoblastic column might present high proliferative potential, but their differentiation, i.e., epithelial–mesenchymal transition, is questionable. In preeclampsia, the expression of epithelial components on the cytotrophoblast surface, such as integrin α_6_β_4_ and E-cadherin, are not downregulated; additionally, the upregulation of markers characteristic of EMT, such as α_1_β_1_ integrin, fails, suggesting that the epithelial–mesenchymal transition is disturbed [23,78,79]. Moreover, the EVT cells in preeclampsia demonstrate the downregulation of HLA-G, which has been implicated in various important processes, including uterine spiral artery remodelling, foetal growth, and the development of foetal–maternal immune tolerance [23]. An excessive inflammatory reaction might also influence the structure and function of the maternal vessels [80]; it is suspected that the initial process of spiral artery transformation, which should begin before the trophoblast reaches the maternal vessels, is inhibited under inflammatory conditions. The wrong preparation of maternal tissues might disturb trophoblast invasion and further artery remodelling. As a result, the placental cells are forced to exist under hypoxic conditions throughout gestation. Studies indicate the presence of low oxygen tension, i.e., about 2% O_2_, in preeclamptic placentas, generating apoptosis in the trophoblast [81]. The ischaemic placenta starts to generate placental debris as well as anti-angiogenic factors, i.e., sFlt1 or sEng, which intensify the chronic maternal inflammation and support the development of oxidative stress. Moreover, the availability and production of placental factors that antagonise pro-antigenic factors, such as vascular endothelial growth factor (VEGF) and placental growth factor (PlGF), is lowered in preeclamptic pregnancies [82,83,84,85]. The depletion in PlGF level, presented as the multiple of the median (MoM), in the maternal blood in the first trimester of gestation is one of the strongest predictors of preeclampsia after week 20 of gestation according to The Fetal Medicine Foundation algorithm [86]. It is believed that the downregulation of proangiogenic factors might promote the dysfunction of maternal endothelial cells, manifesting as a loss of production of endothelial nitric oxide synthase (eNOS) [87] and hence a reduction in the level of nitric oxygen (NO), the major endothelium-derived vasodilator and anti-inflammatory signalling molecule [88]. It is believed that endothelial dysfunction plays a central role in the appearance of hypertension and proteinuria: the most common clinical symptoms observed in preeclampsia [89,90].

## 4. Animal Models of Preeclampsia

The long period between the first event initiating the development of preeclampsia and the resulting clinical symptoms indicates that the development of preeclampsia is a multi-stage process that requires a constellation of events in humans. As such, it is difficult to find an animal model that reflects all of the stages of pathological human gestation; indeed, the period of gestation, the process of implantation, and placental formation as well as hormonal or immunological status vary considerably between animal species and humans. In this sense, all known animal models are therefore incomplete, and even non-human primate animal models presenting spontaneous preeclampsia have some limitations [91]. This restricts the utility of animal models to the investigation of selective fragments of the pathomechanism of preeclampsia [92].

As presented in Table 1, the most commonly used rodents for the study of preeclampsia are mice and rats. Hamsters and guinea pigs are not popular for studies of pregnancy disorders despite demonstrating some common features with human pregnancy, i.e., cytotrophoblast plugs (hamster) or a hemimonochorial placenta (guinea pig). This might be related with the difficulties in the process of measuring blood pressure for these animals. Rats and mice have a long tail, allowing for the non-invasive measurement of blood pressure. Invasive methods, such as telemetric probes, entail surgery with a high risk of mortality due to thrombosis, blood loss, or systemic infection. Moreover, these methods increase the stress experienced by the animal, and the size of the probe has to be matched to the size of the animal, which changes considerably over the course of gestation [93,94]. Additionally, the experimental tools are not readily available for mechanistic analyses of placentation in hamsters and guinea pigs [17], and the downstream laboratory examinations of samples obtained from these animals require reagents dedicated to the studied animals. A number of reagents, e.g., the antibodies used for Western blot or ELISA techniques, do not allow for the examination of guinea pig proteins, requiring the creation and purchase of special reagents.

### 4.1. An Incorrect Trophoblast Invasion Model of Preeclampsia

Although the main source of preeclampsia development is believed to be incorrect trophoblast invasion, it is not currently clear whether this process is the primary defect or a consequence of other processes related to the decidualisation or preparation of maternal vessels for contact with cytotrophoblast [36]. Additionally, no single factor in the maternal blood or uterus has been identified that inhibits cytotrophoblast invasion and maternal spiral artery transformation. Therefore, any insights regarding shallow trophoblast invasion and the incorrect remodelling of uterine vessels obtained after removing the placenta cannot be predicted at the beginning of the study, and they should therefore be regarded merely as side effects of preeclampsia induction in laboratory animals.

The most common preeclamptic animal models presenting insufficient trophoblast invasion are related to the generation of inflammation at the beginning or middle period of pregnancy (Table 1). In these animal models, the inflammatory state is generally generated by a low dose of lipopolysaccharides (LPS) obtained from Gram-negative bacteria, i.e., *Escherichia coli*. In rat models, hypertension is obtained by the intravenous injection of a single dose of LPS (0.5 µg/kg body mass) on the fifth gestational day (GD) [95] or of 1 µg/kg body mass at day 14 [129] or is generated by a single intraperitoneal infusion of a low-dose of LPS (10 µg/kg body mass) at GD 13.5 followed by daily injections of higher doses (40 µg/kg body mass) until GD 16.5 [130]. Similarly, in mouse models, trophoblast invasion impairment and spiral artery remodelling can be induced by daily intraperitoneal LPS treatment (20 µg/kg body mass) from 7.5 to 17.5 [100]; this also generates a preeclampsia-like phenotype.

High blood pressure is typically observed a few hours after the first LPS injection [100,129,130]. Interestingly, hypertension is only observed in the population of pregnant animals; the non-pregnant animals do not respond to the supplementation of LPS with an elevation in blood pressure. It is possible that the inflammation generated by LPS acts as the first signal initiating the pathomechanism of preeclampsia, which is followed by incorrect decidualisation or trophoblast invasion. The impairment of decidualisation and the transformation of maternal uterine vessels may be responses by the foetus/mother to the ongoing inflammatory process in the maternal environment [131]. It is believed that chronic, often asymptomatic, infection might play a significant role in the development of preeclampsia. Therefore, the association between periodontal disease and the outcome of preeclampsia is not surprising. Chronic oral infection predisposes the patient to systematic illnesses, especially those associated with the cardiovascular system; one example is atherosclerosis, which also activates maternal inflammatory mechanisms [132,133]. These mechanisms are also activated by other chronic disorders, including obesity, diabetes, hypertension prior to pregnancy, or autoimmunological diseases, which are known risk factors for the development of preeclampsia in humans.

Inflammation, especially that located in the uterine tissue, might be intensified by the hypoxic conditions dominating in the uterus at the beginning of all pregnancies. This temporally limited hypoxic state, i.e., about 2% O_2_, is necessary for the correct process of trophoblast invasion and proliferation and for the remodelling of the maternal vessels. However, chronic hypoxia seems to be a negative regulator of maternal vessel transformation. Guinea pig mothers maintained in hypoxic conditions, i.e., 10.5% O_2_ from 20 GD until the middle of gestation, demonstrated elevated trophoblastic cell proliferation, but with impaired invasive potential and maternal vessel remodelling [102]. Similar results were observed for populations of pregnant rats maintained in hypoxic conditions, i.e., 10.5% O_2_ between days 6 and 21 of gestation. These rats presented high blood pressure, which increased significantly on day 12 of pregnancy, and demonstrated a higher protein to creatinine ratio in the urine compared to controls. The animals presented the impairment of trophoblast invasion or uteroplacental vessel remodelling and also demonstrated foetal growth restriction [134]. In addition, chronic low-oxygen tension was found to significantly impact the elevation of endothelin-1 level in maternal plasma, one of the strongest vasoconstriction factors secreted both by ischaemic placenta and maternal endothelial cells [134].

The hypothesis that incorrect trophoblast invasion might induce preeclampsia development has also been verified in spontaneously induced preeclampsia animal models, including those based on stroke-prone spontaneously hypertensive rats (SHRSP). These rats demonstrated hypertension before pregnancy and a significant elevation in blood pressure compared to baseline at day 13 of gestation. They also manifested defects in trophoblast invasion and uteroplacental vessel transformation, as indicated by histochemical studies of placentas [99].

Similar to SHRSP rats, BPH/5 mice, i.e., a genetically pre-hypertensive animal model obtained by mating the brother and sister of hypertensive BPH/2 mice, also demonstrate problems with trophoblast invasion and spiral artery remodelling [101]. In addition to the placentation problem, BPH/5 mice also exhibit excessive inflammation in the implantation site, resulting in litters with a low number of pups with low weight [122].

Interestingly, the histochemical results obtained from SHRSP rats and BPH/5 mice are contradicted by another Sprague Dawley rat model derived by mating animals harboring one human renin (hRen) gene or human angiotensinogen gene (hAogen), i.e., genes coding for proteins regulating blood pressure in humans. After mating with a male (hRen), the dams (hAogen) developed hypertension at day 13 of gestation and presented albuminuria; in addition, similar to preeclamptic women, the clinical symptoms of preeclampsia disappeared after gestation [97,98]. The dams also delivered smaller litters with lower mean weight compared to the controls created by mating a normal female rat with a male rat harboring hRen or with a normal male rat. Interestingly, rats presenting the clinical features of preeclampsia demonstrated significantly deeper trophoblast invasion than the controls, determined at GD 18, i.e., on the day of maximal invasion of trophoblasts into the mesometrial triangle. These preeclamptic dams also presented a high rate of fibrinoid deposition and lower numbers of endothelial maternal cells in the uterine spiral arteries; both features are recognised as desirable physiological changes associated with vascular remodelling in both human and rat pregnancies [97].

These findings suggest that the pathomechanism of preeclampsia varies slightly between cases depending on the first signal of activation [135,136,137,138]. It is possible that defects in the regulation of the renin–angiotensin–aldosterone (RAA) system may increase the risk of preeclampsia but via different mechanisms depending on the nature of the defects. In pregnancy, the RAA system not only regulates maternal blood pressure and fluid homeostasis, but it is also believed to play a role in processes associated with the development and functioning of the placenta, such as in angiogenesis, cytotrophoblast proliferation, maternal spiral artery remodelling, placental nutrient transport, and placental hormone secretion [139,140]. Therefore, individual defects in the RAA system could influence the development of either early-onset or late-onset preeclampsia [141]; this could be one of the reasons why defects in trophoblast invasion are not always as evident for late-onset preeclampsia as in cases developing before week 34 of gestation

### 4.2. The Placental Ischaemia in Animal Models for Preeclampsia

**Figure 3:**
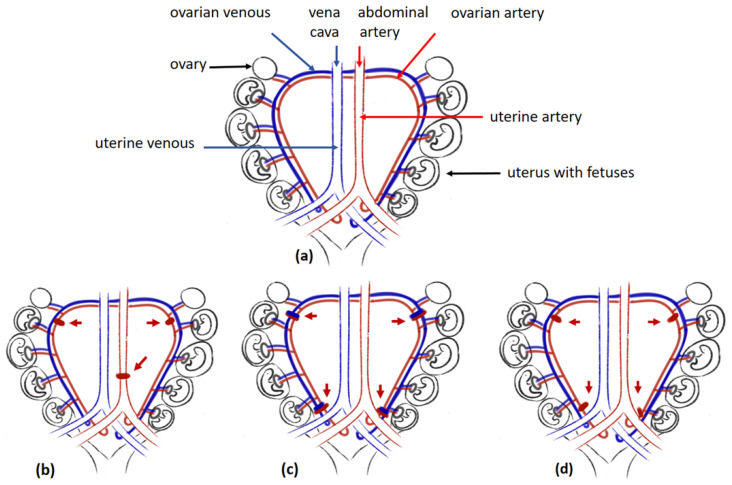
The RUPP models: (**a**) Uterus of rat, (**b**) conventional RUPP model, (**c**) new RUPP model, (**d**) selective RUPP model. Legend: red arrow pointing at silver clips placed around vessels; red clips are placed around the arteries; blue clips are placed around veins.

Although previous models indicate an association between disturbances in trophoblast invasion, maternal spiral remodelling, and preeclampsia development, they do not constitute sufficient proof whether improper placentation, which can lead to chronic placental ischaemia, can fully account for the clinical symptoms of preeclampsia. However, this can be confirmed by animal models based on the mechanical non-complete occlusion of vessels delivering maternal blood to the uteroplacental unit. This reduced uterine perfusion pressure (RUPP) model is characterised by an approximately 40% reduction in uterine perfusion. Depending on the study, vessel occlusion was generated by silver clips or ligation. The three most common locations (Figure 3) were around the aorta right above the iliac bifurcation and around the left and right uterine arcade at the ovarian artery before the first segmental artery to prevent the adaptive increases in blood flow in the ovarian arteries (the conventional RUPP model, Figure 3b) around the uterine arterial and venous branches of the vascular arcade at their ovarian vessels or uterine vessels (the new RUPP model, Figure 3c) or on the ovarian and uterine arteries (the selective RUPP model) (Figure 3d). Additional RUPP model variants are given elsewhere [142]. Controls for all the RUPP animals were created by sham-operated animals or were non-pregnant females that underwent RUPP surgery.

Although the method of occlusion of maternal vessels varies between studies, in most cases, surgery was performed about seven to nine days before the delivery date typical of the species, i.e., about 11.5–14.5 days postcoitum for mice, at about gestational day 14 for rats, or at about day 22 for rabbits [103,105,106,107,143]. Interestingly, while both mouse and rabbit models presented elevated blood pressure 24 h after surgery, hypertension took three or more days to develop in rats, and it was maintained until the day of termination of gestation [105,107,144]. Although RUPP animals develop other clinical features indicative of preeclampsia, e.g., proteinuria, glomerular defects, or foetal growth restriction [73,106,142,144], the precise nature of the changes vary between RUPP variants. For example, among mice treated with a new RUPP model (Figure 3c) in which only some places of ligation were preserved (1) around the ovarian vessels distal to the branches to the ovaries (O,O mouse model) or (2) around the uterine vessels (U,U mouse model), only the latter presented characteristic changes in sFlt1 and PlGF placental gene expression, as observed in preeclamptic women [106].

Similarities can be found between animal models based on conventional RUPP surgery and preeclamptic women with regard to the profile of factors regulating angiogenesis, placental development, and endothelial dysfunction as well as an immunological maternal reaction. This profile includes elevated levels of soluble Flt-1, one of the most important anti-angiogenetic placental-derived factors [103,142,143,145]. Moreover, similar to human preeclamptic pregnancies, ischaemic placentas are known to downregulate PlGF expression; both PIGF and VEGF were found depleted in RUPP rat serum [142,145].

RUPP rats demonstrate a similar serum inflammatory cytokine profile to that of human preeclamptic mothers, with higher levels of IL-6 and TNFα; however, this is not noted in RUPP mice. These cytokines stimulate the production of autoantibodies against the angiotensin II-type receptor (AT1-AA), whose level is also elevated in preeclamptic pregnancies [146,147,148,149].

Although RUPP animals share a number of common features with human preeclamptic mothers, this model is not ideal. A key disadvantage is that as the RUPP model is created by the mechanical occlusion of maternal vessels, it is not suitable for the investigation of the patomechanism of preeclampsia placed before the day of surgery, i.e., generally before days 11.5–14 of gestation in rodents and before day 22 in rabbits. Secondly, some studies indicate that the occlusion of uterine maternal vessels not only inhibits blood flow in the uterus but also in other organs, i.e., heart, kidney, or brain [142]. Finally, this model may not be applicable for testing new drugs whose mechanism of action might be related to increased blood flow to the placenta. However, despite this, the RUPP model delivers unquestionable proof that the ischaemic placenta secretes a number of factors regulating the process of angiogenesis, whose incorrect levels in maternal blood are linked with the pathomechanism of preeclampsia.

### 4.3. Models of Preeclampsia Employing the Incorrect Angiogenesis

RUPP models indicate that the ischaemic placenta produces elevated levels of anti-angiogenic factors, such as sFlt-1 and soluble endoglin 1 (sEng1), and that their levels negatively correlate with those of PlGF and VEGF in maternal circulation [4]. The roles of these anti-angiogenic factors in preeclampsia have been determined by stimulating animals with these agents (Table 1).

The animal models mimicking placental disorders are generated by the infusion of sFlt1 or sFlt1 and sEng1 particles. Intraperitoneal injections of recombinant sFlt1 between days 13 and 18 of gestation were found to generate hypertension in pregnant rats; in addition, treatment with an adenovirus carrying sFlt1 stimulated hypertension in a mouse population at day 8 of gestation [114,150,151]. The stimulated animals also presented features of glomerular damage, with 70% amelioration in the glomerular production of nitrate oxide being observed in some models [113,152]. Interestingly, sFlt1 influences the generation of high blood pressure in both pregnant and non-pregnant animals: non-pregnant Sprague Dawley rats and Balb/c mice treated by adenoviruses carrying the sFlt1 were both found to demonstrate a preeclamptic phenotype [108,113]. These animal models confirm that placentally derived particles such as sFlt1 or sEng play a significant role in the regulation of blood pressure. Moreover, these models are suitable for the investigation of new therapies based on targeting sFlt1 to reduce pregnancy-induced hypertension [153,154,155].

Correct cell–cell communication is essential for ensuring the proper functioning of all organisms. Exosomes, extracellular vehicles, are secreted by most cells to transport various substances, e.g., mRNA, miRNA, DNA, or proteins, for short or long distances [156]. The exosomes secreted into maternal blood by the preeclamptic placenta are sources of anti-angiogenic factors such as sFlt1 and sEng1. Research on C57/BL6 pregnant mice indicates that the exosomes isolated from the serum of preeclamptic women (PE-exosomes) can stimulate hypertension and renal disorders in pregnant animals. These mice also presented lower litter weights, probably as a consequence of the extensive vascular damage caused to the vessels localised within the labyrinth of the placenta [112]. Both sFlt1 and sEng1 were also found to induce severe preeclampsia in Sprague Dawley rats [109]. A significant body of evidence indicates that sFlt1 neutralises proangiogenic factors circulating in maternal blood, such as the free forms of VEGF and PlGF; in addition, sEng1 not only negatively regulates angiogenesis but also increases the permeability of the maternal vessels and blocks the activation of endothelial nitric oxide synthase (eNOS), downregulating the production of nitric oxide, one of the strongest vasodilators [109].

The inhibition of angiogenesis was found to influence the development of preeclampsia in a group of pregnant Sprague Dawley rats treated by Suramin, an angiogenesis inhibitor antagonizing VEGF, platelet-derived growth factor, or fibroblast growth factor [110]. The rats demonstrated hypertension accompanied by placental dysfunction, renal failure, and reductions in foetal body mass. Interestingly, the Suramin-treated rats demonstrated over 50% lower sFlt1 levels compared to the controls. This may be the reason for the smaller litter numbers carried by the treated rats and thus the smaller number of placentas secreting sFlt1 compared to the controls [110]. These findings might also suggest that other unidentified maternal factors appearing in maternal blood might make preeclampsia development more likely. Indeed, although preeclamptic women demonstrate the classical pattern of high levels of circulating sFlt1 and low levels of free PlGF, some studies suggest that preeclampsia is also associated with low sFlt1 and high PlGF levels [157]. It is possible that the placental cells of these women secrete other factors that deplete VEGF levels and thus disturb angiogenesis. Treatment with sFlt1 inhibitors will certainly be ineffective in such populations; therefore, models based on Suramin treatment might be a promising basis for designing personal therapies for these rare groups of preeclamptic women.

### 4.4. Inflammatory Factors Exert Preeclampsia in Animal Models

Preeclampsia is clearly related to the activation of immunological systems and is manifested by increases in TNFα, IL6, or IL1 levels in maternal circulation. Such inflammation accompanies preeclampsia from the beginning of gestation and continues throughout the course of preeclamptic pregnancy. A number of studies indicate that maternal pre-pregnancy disorders associated with inflammation, such as obesity, diabetes, or autoimmunological diseases, predispose one to the development of preeclampsia. Early inflammation has been confirmed to induce hypertension in animal models treated by LPS at the beginning of gestation (Table 1). The treated rats and mice developed hypertension and trophoblast invasion disorders, as discussed in the previous section, Section 4.1. “An incorrect trophoblast invasion model of preeclampsia”.

It seems that inflammation does not only influence the development of preeclampsia, but the mechanism of its action depends the strength and the time of its initiation. A significant body of evidence indicates that high levels of sFlt1 not only result from placental ischaemia but also from the response of placental cells to inflammation. Sprague Dawley rats administered 50 ng/day of TNFα for five days beginning from day 14 of gestation presented a significant increase in the sFlt1 level in maternal blood and higher mean arterial pressure (MAP) in comparison to not-treated controls. This elevated sFlt1 level, caused by chronic infusion of TNFα, was reduced after maternal injections of a soluble TNFα receptor [158].

Interestingly, the infusion of a single dose of TNFα after the middle of gestation, i.e., at day 13.5 of gestation-induced placental hypoxia in C57BL/6JArc mice, resulted in the elevated expression of hypoxia inducible factor 1 (HIF1) in all sections of the mouse placentas, i.e., the labyrinth, junctional zone, and decidua. Although significant expression of the gene coding for Flt1 was noticed in ischaemic placentas, surprisingly, the levels of Flt1 in dam serum fell, albeit insignificantly [120].

The injection of inflammatory factors into pregnant animals not only influences the placenta, but also forces the maternal organism to generate agents similar to those observed in preeclamptic women. Daily infusions of TNFα to rats between days 14 and 19gestation induced the significant generation of AT1-AA antibodies, which are responsible for the activation of the angiotensin II type I (AT1R) receptor, which is found during vasoconstriction, salt-water retention, and aldosterone secretion [149,159]. Elevated AT1-AA antibody levels are also typical of human pregnancies complicated by preeclampsia or abnormal uterine perfusion. Interestingly, the chronic infusion of TNFα into non-pregnant rats did not increase the production of AT1-AA antibodies, suggesting that the elevation in AT1-AA occurring in maternal serum in response to inflammation is restricted to pregnancy [149]. Similarly, a relationship between pregnancy-induced hypertension and high AT1-AA levels was observed in Sprague Dawley rats after IL6 infusion, suggesting that the production of these antibodies depends on the presence of unwanted inflammation in pregnancies [118].

The reason why AT1-AA generation is augmented in pregnancies complicated by inflammation remains a mystery. Nevertheless, it is believed that these antibodies and various inflammatory factors have a direct impact on the dysregulation of the maternal endothelium. AT1-AA antibodies mainly support the process of vasoconstriction by stimulating endothelial cells to produce endothelin-1 [160]. Similar effects, such as the elevated expression of preproendothelin mRNA in the kidney, placenta, and aorta, were obtained by daily the infusion of TNFα for five days beginning from day 14 of gestation [161]. Moreover, TNFα is perceived as a mediator in the vascular contraction influencing endothelial dysfunction. It inhibits acetylcholine- and bradykinin-induced vascular relaxation as well as nitrate production in endothelial cells, especially in pregnant rats [162]. Although animal models based on the supplementation of inflammatory factors indicate that both chronic and acute inflammation play significant roles in the development of the clinical features of preeclampsia, these models are not good candidates for determining whether endothelial dysfunction, a consequence of inflammation, is sufficient to generate the clinical symptoms of the disease.

### 4.5. Endothelial Dysfunction: The Last Step in the Generation of Preeclampsia

A significant body of evidence indicates that endothelial dysfunction has a key influence on the development of the preeclamptic phenotype. It is known that the vasodilatory propriety of endothelial cells influences the regulation of blood pressure, especially during pregnancy, which is characterised by high cardiac output, blood volume, and heart rate compared to a pre-pregnancy baseline but normal or slightly lower blood pressure. The most important regulator of blood pressure is nitric oxide (NO), which is produced in endothelial cells from L-arginine by enzyme endothelial nitric oxide synthase (eNOS). The eNOS knockout mice present the preeclamptic phenotype; however, hypertension is recognised both before and during pregnancy [128,138,163], and proteinuria develops with late gestation, i.e., at 18.5 GD [128]. Although the development of hypertension induced by pregnancy is faint, these mice present other clinical features typical of preeclampsia. The most commonly reported findings are foetal growth restriction and reduction in uterine blood flow. Moreover, hypoxia and reduced nutrient transport capacity are characteristic conditions observed in the placentas of mice lacking genes coding for endothelial nitric oxide synthase [128,164]. The production of NO could be blocked by L-arginine analogues such as nitro-L-arginine methyl ester (L-NAME), which causes NO deficiency by inhibiting NOS activity [123]. The reaction of animal models depends on the mode of L-NAME supplementation: one study on a rat model found that L-NAME treatment at the beginning of gestation, i.e., between days 9 and 11, resulted in a lower rise in mean blood pressure (MAP) compared to later treatment between days 18 and 20 [165]. This suggests that the endothelium has a significant impact on the regulation of blood pressure, especially in the last trimester of pregnancy. This period of gestation is characterised by high blood volume in maternal circulation; as such, the regulation of vascular tone might play a significant role in maintaining correct blood pressure. Numerous studies indicate that supplementation with L-NAME induces hypertension and proteinuria in pregnant rodents and rabbits [123,166,167,168,169], and some animals present additional symptoms, i.e., thrombocytopenia and intrauterine growth restriction, which are indicative of preeclampsia in humans [123]. Moreover, rats treated with L-NAME demonstrate a higher sFlt1 to PlGF ratio in maternal serum than untreated controls [124]; however, this ratio was insignificantly elevated for mice treated with L-NAME [170].

The offspring born from mothers treated with L-NAME during pregnancy were found to suffer from hypotension for the first two weeks of their postnatal life [171]. A similar observation was noted in the first hours after delivery for human infants born from preeclamptic pregnancies [172]. Interestingly, gravid and non-pregnant rats demonstrated a similar response to L-NAME infusion [173]: although both groups demonstrate a preeclampsia-like phenotype, the presence of hypertension and proteinuria in virgins contradicts the main assumption of the diagnosis of preeclampsia, as it is reserved for the gestational period. This suggests that endothelial dysfunction is necessary to provoke the development of the clinical symptoms of preeclampsia. Unfortunately, L-NAME animal models are not suitable for providing a full picture of this disease, especially the aspects related with placental dysfunction. Indeed, the placentas obtained from a murine L-NAME model did not present any alternations in the labyrinth or junctional zones or in the maternal deciduae. The only changes were observed for sinuses, which were narrower than those of the control dams [170].

The mode of induction of hypertension for these models is also questionable. Although pregnancies not complicated by hypertension demonstrate an amelioration in NO production, the association between the downregulation of NO and preeclampsia remains ambiguous [148,174]. However, the L-NAME model is a good candidate model for investigating potential drugs that might be used to modulate the disturbed pathways in the endothelium of preeclamptic women [175]. Such drugs might be desirable for treating women manifesting the whole spectrum of preeclamptic symptoms; indeed, by modifying the disturbed pathways in the maternal endothelium, it may be possible to suppress disease-related symptoms and to thus increase the length of gestation, giving the foetus more time to thrive.

## 5. Conclusions

To summarise, the types of gestation observed in the presented animal models differed from human gestation. Even rodents and rabbits were found to differ from one another, e.g., in the time of duration of gestation, the time of implantation, the time and depth of trophoblast invasion, and the placental architecture (Table 2).

All of these differences between human and rodents or between human and rabbits should be considered when choosing the best model for an experiment. Models intended for researching the primary reason for preeclampsia development will be strongly influenced by the depth of trophoblast invasion, the remodelling of uterine vessels, or decidualisation as well as by the time for the initiation of animal stimulation or the termination of pregnancy.

For example, it is important to note that trophoblast invasion dominates at the beginning of gestation in humans, but for rodents, it begins in the middle of gestation and is completed a few days before delivery. Similarly, while the influx of maternal blood into the placental lacunae starts at the beginning of pregnancy in humans, it takes place at about in mid-gestation in rodents. Therefore, the effect of placental hypoxia should be examined after maternal blood influx in the studied animals.

Models based on inflammation are more universal, as they allow for the observations of various pathomechanistic aspects of preeclampsia, which change over the course of gestation. As such, these models could be still improved; indeed, it is clear that inflammation accompanies both non-complicated and preeclamptic pregnancies from the first day. It is also known that in preeclampsia, inflammation is not ameliorated during gestation; in fact, it influences the appearance of the clinical symptoms of preeclampsia after week 20. In contrast to humans, animal models present an increase in blood pressure within the first 24 h from the first contact with the inflammatory factor or inductor of inflammation, e.g., LPS.

Irrespective of the time of occurrence of hypertension, animal models seem to be a good candidate for testing potential preventive drugs that might protect against inflammatory-derivative placental ischaemia or maternal endothelium dysfunction when administered before pregnancy or at the beginning. In this respect, L-NAME models seem to be good candidates to determine the association between the pathological pathways taking place in the maternal endothelium and the occurrence of symptoms of preeclampsia. Furthermore, certain models, e.g., L-NAME and sFlt-1, are also useful for long-term studies presenting maternal or foetal postnatal complications of the cardiovascular system [94,168,194,195,196]. As demonstrated above, while an animal model can provide insight into an individual aspect of preeclampsia, no single model can currently represent the full complexity of the etiology of preeclampsia.

## Figures and Tables

**Table 1:** The generation of preeclampsia by the induction of various aspects of the disease in animal models.

Species	Inducing Factor	Time of PE Induction	HA	P or A/C Ratio	Ref
Incorrect trophoblast invasion					
Rat	0.5 µg/kg LPS	5 GD	+	+	[95,96]
Rat	Transgenic rats hAngiotensinogen female x hRenin male	spontaneously	+	+	[97,98]
Rat	Stroke-prone spontaneously hypertensive rat (SHRSP)	spontaneously	+	+	[99]
Mouse	LPS 20 µg/kg	7.5–17.5 GD	+	+	[100]
Mouse	BPH5 mouse	spontaneously	+	+	[101]
Guinea pig	Exposure to hypoxia 10.5% O_2_	28–30 GD up to term	+	ns	[102]
Placental ischaemia					
Rat	RUPP model	14 GD	+	-, ns	[103,104]
Rat	Selective RUPP model	14 GD	+	ns	[105]
Mouse	RUPP model	14.5 GD	+	+	[106]
Rabbit	RUPP model	25 GD	+	-	[107]
Incorrect angiogenesis					
Rat	Injection—adenovirus expressing sFlt-1	8 or 9 GD	+	+	[108]
Rat	Injection—adenovirus sFtl-1 and/or sEng	8 or 9 GD	+	+	[109]
Rat	Injection—Suramin (an inhibitor of angiogenesis)	10 and 11 GD	+	-	[110]
Mouse	Injection—adenovirus expressing stabilised HIF-1α	8 GD	+	+	[111]
Mouse	Injection—exosomes of preeclamptic women	5.5; 10.5; 15.5 GD	+	+	[112]
Mouse	Injection—adenovirus carrying sFlt-1	4; 8; 9 GD	+	+	[113]
Mouse	Injection—adenovirus carrying sFlt-1	8 GD	+	+	[114]
Mouse	Injection into blastocyst lentivirus carrying human sFlt-1	in vitro	+	+	[115]
Inflammation					
Rat	TNFα infusion	14–19 GD	+	ns	[116]
Rat	TNFα infusion	14 GD	+	-	[117]
Rat	IL6 infusion	14–19 GD	+	-	[118]
Mouse	Injection of purified IgG from PE women	13 and 14 GD	+	+	[119]
Mouse	TNFα injection	13 GD	+	+	[120]
Mouse	IL10 deficient mouse	spontaneously	+	+	[121]
Mouse	BPH5 mouse	spontaneously	+	+	[122]
Endothelial dysfunction					
Rat	L-NAME in drinking water	8–19 GD	+	+	[123]
Rat	L-NAME injection	9-20 GD or 10-20 GD	+	+	[124]
Rat	AT1-AA antibody injection	13 and 14 GD	+	+	[125]
Mouse	L-NAME injection	7–18 GD	+	+	[126]
Mouse	AT1-AA antibody injection	13 GD	+	+	[127]
Mouse	eNOS knockout mice	spontaneously	+	+	[128]

PE—preeclampsia; GD—gestational day; HA—hypertension; P or A/C—proteinuria or albumin to creatinine ratio in urine; LPS—lipopolysaccharide; RUPP—reduced uterine perfusion; sFlt-1—soluble fms-like tyrosine kinase 1; sEng—soluble endoglin-1; TNFα—tumour necrosis factor alpha; IL6—interleukin 6; IL10—interleukin 10; L-NAME—N-nitro-L-arginine methyl ester, an inhibitor of NO synthase (NOS); AT_1_-AAs antibodies—antibodies against the angiotensin II receptor type 1a (AT_1_ receptor) localised on endothelial cells; IgG—immunoglobin G; BPH5—high blood pressure (BPH)/5 mouse; eNOS—endothelial nitric oxide synthase. +—present, -—not measured, ns—non significant differences between studied groups.

**Table 2:** Comparison of pregnancy-related features between humans and small mammals, i.e., rodents and rabbits.

	Human	Mouse	Rat	Hamster	Guinea Pig	Rabbit	Ref
Gestation days	280 days	20–21 days	About 21 days	About 16 days	59–72 days	28–31 days	[14,55,70,176,177]
Maternal recognition of pregnancy	Luteotrophic factor (choronic gonadotrophin)	Vaginal stimulation + luteotrophic factor (pituitary prolactin)	Vaginal stimulation + luteotrophic factor (pituitary prolactin)	Vaginal stimulation + luteotrophic factor (pituitary prolactin)	Luteotrophic factor from pituitary	Vaginal stimulation + luteotrophic factor (oestrogen)	[17,178,179,180]
Signal for decidualization	Hormonal stimulus	Hormonal and implantation stimulus	Hormonal and implantation stimulus	Hormonal and implantation stimulus	Hormonal and implantation stimulus	Hormonal and implantation stimulus	[17]
Implantation	5–6 after ovulation	4–5 GD	5–6 GD	4–5 GD	7–8 GD	7–9 GD	[12,15,59,176,181,182]
Model of implantation	Interstitial	Eccentric/ interstitial	Eccentric/ interstitial	Eccentric/ interstitial	Interstitial	Superficial	[9,183]
Trophoblast invasion	Post implantation—9 weeks (interstitial) 9–22 weeks (endovascular)	14 GD	12.5–13.5 GD	Before 12 GD	10–30 GD	8–9 GD	[17,39,58,60,184,185,186,187,188,189]
Way of invasion	I/E	I	I/E	E	I/E		[36]
Deepness of trophoblast invasion	Myometrial	Decidual	Mesometrium	Mesometrium	Mesometrium and far beyond uterus	Mesometrium	[36,49]
Trophoblastic plug	+	-	-	+	-	-	[50]
Spiral arteries remodelling	Before pregnancy	8–12 GD	6.5–13.5 GD	5–6 GD	Before 30 GD	About 8 GD	[3,17,38,57,58,60]
Maternal blood influx into sinus/lacunae	10–12 W	9.5–14.5 GD	12–15 GD	12 GD	About 18 GD	About 10 GD	[51,52,53,54,55,56,186,190,191,192]
Type of foetal/ maternal barrier	Haemomonochorial (1) Foetal blood (2) FE (3) BM (4) **SYN** (5) Maternal blood	Haemotrichorial (1) Foetal blood (2) FE (3) BM (4) **SYN** (5) **SYN** (6) **CYT** (7) Maternal blood	Haemotrichorial (1) Foetal blood (2) FE (3) BM (4) **SYN** (5) **SYN** (6) **CYT** (7) Maternal blood	Haemotrichorial (1) Foetal blood (2) FE (3) BM (4) **SYN** (5) **SYN** (6) **CYT** (7) Maternal blood	Haemomonochorial (1) Foetal blood (2) FE (3) BM (4) **SYN** (5) Maternal blood	Haemodichorial (1) Foetal blood (2) FE (3) BM (4) **CYT** (5) **SYN** (6) Maternal blood	[9,71]
Modification of chorionic surface	Villi	Labyrinth	Labyrinth	Labyrinth	Labyrinth	Labyrinth	[9,70]
Placenta Foetal side	(1) Villous trophoblast—placental–foetal interface. (2) Extravillous trophoblast cells—uterine–placental interface.	(1) Labyrinth corresponding to human villous trophoblast. (2) Junctional zone (known as basal zone) corresponding to human extravillous trophoblast.	(1) Labyrinth corresponding to human villous trophoblast. (2) Junctional zone (known as basal zone) corresponding to human extravillous trophoblast.	Labyrinth corresponding to human villous trophoblast and including (2) junctional zone (known as basal zone) corresponding to human extravillous trophoblast.	(1) Main placenta, including: (a) labyrinth; (b) interlobium; (c) yolk sac placenta. (2) Placenta-seam, including: (a) Subplacenta. (b) Junctional zone.	(1) Labyrinth corresponding to human villous trophoblast and (2) junctional zone.	[17,33,50,70]
Placenta Maternal side	(1) Maternal decidua. (2) Maternal myometrium	(1) Mesometrial decidua. (2) Mesometrial triangle including maternal gland.	(1) Mesometrial decidua. (2) Mesometrial triangle including maternal gland.	(1) Mesometrial decidua. (2) Mesometrial triangle including maternal gland.	(1) Decidua. (2) Mesometrium.	(1) Deciua including necrosis zone and separation zone). (2) Mesometrium.	[33]
Shape of placenta	Discoid	Discoid	Discoid	Discoid	Discoid	Bidiscoid	[9,33]
Yolk sac placentation	12 weeks/20 weeks	Inverted/at term	Inverted/at term	Inverted/at term	Inverted/at term	Inverted/at term	[193]

Legend: GD—gestational day; CYT—cytotrophoblast; SYN—syncytiotrophoblast; FE—foetal endothelium; I—interstitial type of trophoblast invasion; E—endovascular type of trophoblast invasion; in the row “Placenta. Foetal side”, numbers 1 and 2 describe the distance of the placental structure from the maternal side; the structure with number 2 is closer to the maternal side of the placenta. In row “type of foetal/maternal barrier” the numbers represent the layers of the barrier from the foetal to the maternal side in the placenta. In row “Trophoblastic plug”—lack of trophoblastic plug, +—presence of trophoblastic plug.

## Data Availability

Not applicable.
